# *Anopheles barbirostris* in Indonesia: A more complex metapopulation than expected

**DOI:** 10.1371/journal.pone.0321707

**Published:** 2025-11-10

**Authors:** Tri Baskoro Tunggul Satoto, Triwibowo Ambar Garjito, Shinta Shinta, Soleman Landi, Roger Frutos, Sylvie Manguin

**Affiliations:** 1 Department of Parasitology, Faculty of Medicine, Public Health and Nursing, Gadjah Mada University, Yogyakarta, Indonesia; 2 Research Center for Public Health and Nutrition, National Research and Innovation Agency, Indonesia; 3 Public health faculty, Nusa Cendana University, East Nusa Tenggara,; 4 Cirad, UMR 17, Intertryp, Montpellier, France; 5 Faculty of Medicine-Ramathibodi Hospital, Mahidol University, Bangkok, Thailand; 6 Department of Health, Faculty of Vocational Studies, Universitas Airlangga, Surabaya, Indonesia; 7 School of Public Health, Xiamen University, Xiamen, China; 8 HSM, University of Montpellier, CNRS, IRD, Montpellier, France; Clinton Health Access Initiative, UNITED STATES OF AMERICA

## Abstract

*Anopheles barbirostris*, a member of the Barbirostris Subgroup in the *Anopheles* genus, comprises a complex of species in South and Southeast Asia. This *An. barbirostris* complex includes eight species such as *An. barbirostris sensu stricto* (s.s.), *An. campestris*, *An. dissidens*, *An. donaldi*, *An. saeungae*, *An. sarpangensis*, *An. vanderwulpi*, and *An. wejchoochotei*. This study employed molecular markers, including ITS2 and COI genes, to investigate the phylogenetic relationships within *An. barbirostris sensu lato* (s.l.) populations from various locations in Indonesia. The analysis reveals the presence of nine distinct populations within this complex, including the first report of *An. wejchoochotei* in North Sulawesi and a unique Barbirostris population in Magelang, Central Java. This makes it a more complex metapopulation than previously thought. These findings provide critical insights into the diversity of malaria and lymphatic filariasis vectors in Indonesia. Understanding the complex structure of the *An. barbirostris* populations and their genetic diversity will be useful for effective vector control and disease elimination strategies.

## Introduction

*Anopheles barbirostris* is currently identified as a species within the Barbirostris Subgroup of the Subgenus *Anopheles* [1,2]. This subgroup is composed of 11 species, including six that are differentiated by distinct morphological characters, such as *An. barbirostris, An. campestris, An. donaldi, An. franciscoi, An. hodgkini*, and *An. pollicaris* [3,4]*. Anopheles barbirostris sensu lato* (s.l*.*) has been regarded as a species complex since 2001 according to cytogenetics and molecular characteristics [2,5,6]. This *An. barbirostris* complex includes eight species such as *An. barbirostris*, *An. campestris*, *An. dissidens*, *An. donaldi*, *An. saeungae*, *An. sarpangensis*, *An. vanderwulpi*, and *An. wejchoochotei* [2,4,6,7]. This species complex is broadly distributed in South and Southeast Asia, as in Indonesia, Thailand, Malaysia, Timor Leste, Vietnam, Myanmar, Cambodia, Sri Lanka, Nepal, Bhutan, Bangladesh, and China [3,4,8–13]. In Indonesia, *An. barbirostris* s.l. has been reported in Sumatra, Java, Kalimantan, Sulawesi, Bali, Lesser Sunda Islands, and North Maluku (Buru Island). However, this species complex has never been reported in the Maluku and Papua regions [9,14].

Prior to the implementation of molecular analyses, *An. barbirostris* s.l. was regarded as comprising 4 distinct cytological forms [15]. The first evidence of *An. barbirostris* as a species complex was formally reported as W, X, and Z forms based on mtDNA Cytochrome Oxidase I gene (COI) [5]. Subsequent molecular phylogenetic analyses of *An. barbirostris* s.l. based on mtDNA COI and rDNA ITS2 have revealed the presence of several sympatric clades in Sumatra and Java [3]. More recently, based on integrated morphological and molecular (COI and ITS2) characterization, four species within the Barbirostris complex have been reported in Indonesia: *An. barbirostris* in Java and Kalimantan, *An. vanderwulpi* in Java and Sumatra*, An. saeungae* in Sumatra, and an unknown form of *An. barbirostris* in Sulawesi [3,8,16–18].

*Anopheles barbirostris* s.l. has been considered an important malaria and lymphatic filariasis vector due to its anthropophilic behavior in Lesser Sunda Islands and Sulawesi [19–21]. *Anopheles barbirostris* s.l was first reported as a malaria vector in 1939 in South Sulawesi [22]. The role of this taxon as an important malaria vector was also reported in several locations in the Lesser Sunda Islands (Lombok, Flores, and Adonara Islands), Northern Sulawesi (Meras and Tomohon), and Central Sulawesi [23]. Both *Plasmodium falciparum* and *Plasmodium vivax* infections were detected in all of these areas [19,21,24,25]. *Anopheles barbirostris* s.l. has also been confirmed as a Malayan filaria vector in Sulawesi and as a Timor filaria vector in East Nusa Tenggara [26,27]. However, while present in Sumatra, Java, Kalimantan, West Nusa Tenggara, and Maluku, *An. barbirostris* s.l. has not been confirmed as malaria and/or lymphatic filariasis vector in these areas due to its mainly zoophilic behavior [9]. Therefore, it is of upmost importance to identify the species of the Barbirostris complex to evaluate their behavior and their involvement in parasite transmission.

In this study, we used the internal transcribed spacer 2 (ITS2), a ribosomal DNA (rDNA) region, and the cytochrome oxidase I (COI) mitochondrial gene to characterize the phylogenetic relationships of the *An. barbirostris* s.l*.* samples collected from different locations in Indonesia. This information is a first but important step towards the understanding of local malaria transmission dynamics and the design of appropriate vector control strategies within the national malaria elimination program.

## Materials and methods

### Mosquito field collections and species identification

**Fig 1:**
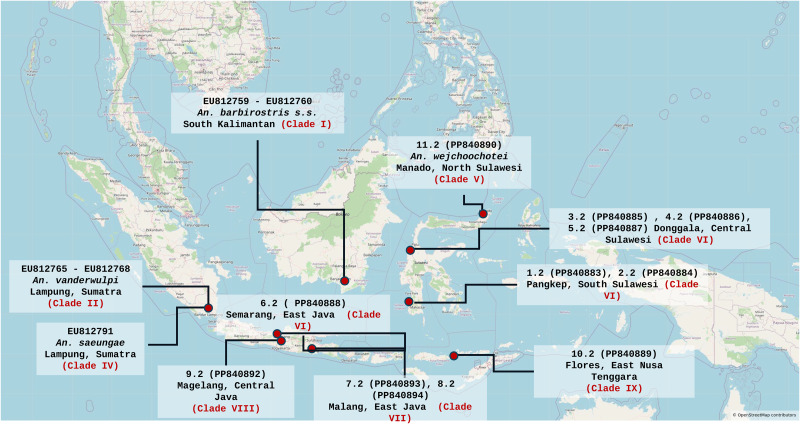
Map of the *An. barbirostris* s.l. associated to 8 clades (I-II, and IV-IX) based on the ITS2 sequences, and locations of the sampling sites (red spots) in Indonesia. Contains information from OpenStreetMap and the OpenStreetMap Foundation, which is made available under the Open Database License.

A total of 243 adult mosquitoes were collected from the field using human-landing collection (HLC) and cattle-baited trap methods in the provinces of North Sulawesi, Central Sulawesi, South Sulawesi, Central Java, East Java, and East Nusa Tenggara (Table 1, Fig 1). Most of the mosquitoes were prepared for storage as dry collections for morphological study purposes, and 17 specimens representing each study site were allowed to proceed for molecular analysis. All specimens were initially identified as *An. barbirostris* s.l. based on standard morphological adult mosquito identification keys for Indonesia [28,29]. These mosquitoes were labeled according to locality and date, and kept individually in 1.5 ml tubes under dry conditions using silica gel until molecular analysis was performed.

**Table 1:** Sampling localities and specimens of the *Anopheles barbirostris* complex.

Sample code	Province	Location	Malaria vector	GenBank ID (ITS2)	GenBank ID (COI)
1-2	South Sulawesi	Pangkajene Kepulauan	Yes	X	X
2−2	South Sulawesi	Pangkajene Kepulauan	Yes	X	X
3−2	Central Sulawesi	Donggala	Yes	X	X
4−2	Central Sulawesi	Donggala	Yes	X	X
5−2	Central Sulawesi	Donggala	Yes	X	X
6−2	Central Java	Semarang-Salaman- Jambu	No	X	X
7−2	East Java	Malang	No	X	X
8−2	East Java	Malang	No	X	X
9−2	Central Java	Magelang	No	X	X
10−2	East Nusa Tenggara	Flores	Yes	X	X
11−2	North Sulawesi	Manado	Yes	X	X
JAVA-J7	Central Java	Semarang	No		X
JAVAS8	Central Java	Magelang	No		X
JAVAJ10	Central Java	Semarang	No		X
FLORES-S1	East Nusa Tenggara	Flores	Yes		X
FLORES-S3	East Nusa Tenggara	Flores	Yes		X
FLORES-R8	East Nusa Tenggara	Flores	Yes		X

### rDNA-ITS2 and mtDNA-COI gene amplification and sequencing analysis

The genomic DNA was extracted from individual mosquitoes using a DNeasy^®^ Blood & Tissue Kit (Qiagen, Hilden, Germany) based on the manufacturer’s protocol. The rDNA-ITS2 amplification was performed with universal primers ITS2-F (5’-GGT GGT GAC TTT CAA GTTC −3’) and ITS2-R (5’-TAT GCT TAA ATT TAG TTT GTA G −3’). PCR reactions were performed using GoTaq^®^ Green Master Mix (Promega Madison, WI, USA). PCR thermocycling conditions for ITS2 were as follows: 95°C for 5 min; followed by 26 cycles of denaturation at 94°C for 1 min, annealing at 55°C for 1 min and elongation at 72°C for 1 min; and followed by a final extension step at 72°C for 7 min. The mtDNA-COI was amplified using primer CI-N-2087 (5’-AAT TTC GGT CAG TTA ATA ATA TAG-3’) and TY-J-1460 (5’-TAC AAT TTA TCG CCT AAA CTT CAG CC-3’) [30]. PCR was also carried out using GoTaq^®^ Green Master Mix (Promega Madison, WI, USA). The mtDNA-COI amplification comprised initial denaturation at 94°C for 1 min followed by five cycles of 94°C for 30 s, 45°C for 40 s, and 72°C for 1 min. This process was then followed by 35 cycles of 94°C for 30 s, 55°C for 40 s, 72 for 1 min, and a final extension at 72°C for 10 min. All PCR reactions were performed in an Applied Biosystems SimpliAmp thermal cycler. After PCR amplification, products were separated by 1.5% agarose gel electrophoresis and stained with SYBR^®^ safe DNA gel stain (Invitrogen, Carlsbad, CA, USA). The amplification products were purified using Applied Biosystems ExoSAP-IT^TM^ (Thermo Fisher Scientific, Vilnius, Lithuania) and sequenced using the primers listed above with an Applied Biosystems Big Dye^TM^ Terminator version 3.1 Sequencing Kit (Life Technologies Cooperation, Austin, TX, USA). Cycle sequencing products were edited using Sequencing Analysis v5.2. (Applied Biosystems), and were aligned using ClustalW 1.6 in Mega X version 10.2.2. The sequences were compared with the available sequences of *Anopheles barbirostris* in the NCBI GenBank database. The maximum likelihood method with the General Time Reversible (GTR)+1 model was constructed in Mega X version 10.2.2. *Anopheles barbumbrosus*, of the Barbirostris Group, was used as an outgroup in the phylogenetic trees. Bootstraps were tested with 1,000 replicates to assess the reliability of the trees. Variations within and between species were measured by the pairwise distance (p-distance) method in MegaX version 10.2.2. Sequences were deposited in NCBI GenBank with the following accession numbers: PP840883-PP840889, PP840890, PP840892-PP840894. Species delimitation was conducting on ITS2 using the PTP web server (https://species.h-its.org/ptp/). Delimitation was calculated by two different methods: Maximum Likelihood and Bayesian analysis under the following conditions: MCMC Generation 100000, Thinning 100, Burning 0.1 and Seed 123. The MCMC chains were tested for positive convergence.

## Results

### Phylogenetic analysis of the Internal Transcribed Spacer 2 (ITS2)

**Fig 2:**
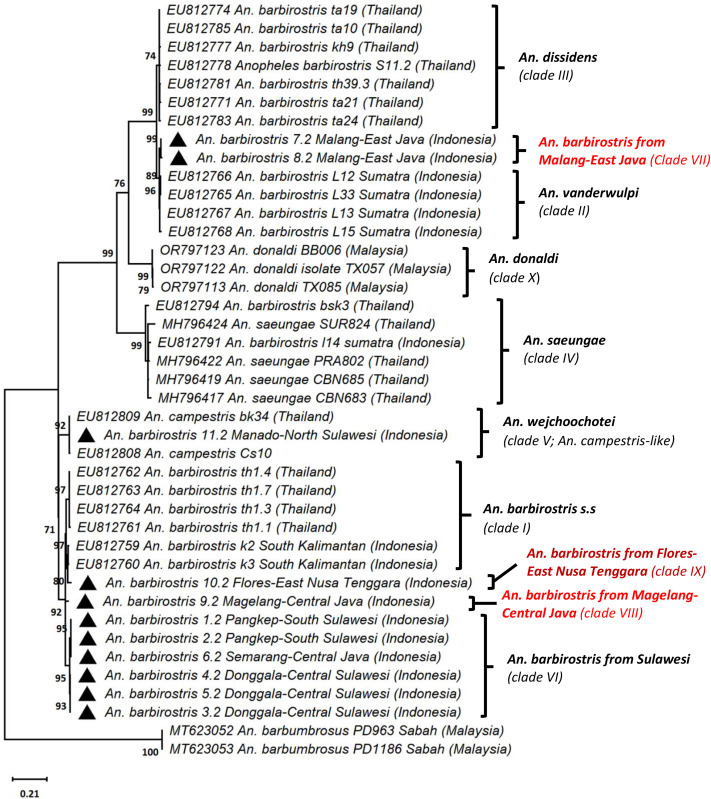
Phylogenetic analysis of the ITS2 sequences of *An. barbirostris* complex. Tree rooted using *Anopheles barbumbrosus* as outgroup. The field collected samples are identified with black triangle. The phylogenetic tree was constructed using the maximum likelihood (ML) method with the Kimura −2 evolutionary model in Mega X. Bootstraps were tested at 1,000 replicates.

The analysis of the rDNA ITS2 sequences of the *An. barbirostris* samples from this work and reference sequences available from GenBank revealed that eight populations of *An. barbirostris* could be identified in Indonesia (Fig 2). The rDNA ITS2 marker, which is not a coding sequence, is a very good marker for phylogeny in particular at the specific level. It is highly conserved and, therefore, a significant branching (i.e., a significantly high bootstrap) is indicative of a different species whereas low bootstrap would rather indicate the presence of populations from a same metapopulation, i.e., same species. These samples include *An. barbirostris* from South Kalimantan (EU812759-EU812760 – clade I), *An. vanderwulpi* from Lampung-Sumatra (EU812765-EU812768 – clade II), *An. saeungae* from Lampung-Sumatra (EU812791 – clade IV), *An. wejchoochotei* from Manado-North Sulawesi (sample 11.2 – clade V), *An. barbirostris* from South (samples 1.2, 2.2) and Central Sulawesi (samples 4.2, 5.2), and Central Java (sample 6.2) all of clade VI, *An. barbirostris* from Malang-East Java (samples 7.2–8.2 – clade VII), *An. barbirostris* from Magelang-Central Java (sample 9.2 – clade VIII), and *An. barbirostris* from Flores-East Nusa Tenggara (sample 10.2 – clade IX). The ITS2 of *An. barbirostris* samples from Malang-East Java (samples 7.2, 8.2 – clade VII) differ from *An. vanderwulpi* from Sumatra (EU812765- EU812768 – clade II) by 1.3–2.7%. High conservation and 100% genetic similarity were identified among samples from Pangkep, South Sulawesi (samples 1.2, 2.2), samples from Semarang-Central Java (sample 6.2), and those from Donggala-Central Sulawesi (samples 4.2, 5.2), all belonging to clade VI. The sample of *An. barbirostris* from Manado, North Sulawesi (sample 11.2 – clade V) displayed 100% ITS2 identity with *An. wejchoochotei* from Thailand (AB971307, AB971309 – clade V) and also showed 99.7% genetic similarity with *An. campestris* from Thailand (EU812808-EU812809 – clade V). The Sulawesi samples (1.2, 2.2, 4.2, 5.2 – clade VI) and Central Java sample (6.2 – clade VI) displayed 0% internal group distance and distances of 3.2–4.3% with *An. barbirostris* from Magelang-Central Java (sample 10.2 – clade VIII), 0.1–2.4% with *An. barbirostris* from South Kalimantan (EU812759-EU812760 – clade I), 3.7–4.3% with *An. barbirostris* from Flores, East Nusa Tenggara (sample 9.2 – clade IX), and 4.5–5.6% with *An. barbirostris* from Thailand (EU812761-EU812764 – clade I). Furthermore, the distances between *An. barbirostris* from Lampung-West Sumatra (EU812791 – clade IV) and *An. saeungae* from Thailand (MH796417, MH796419, MH796422, MH796424, EU812794 – clade IV) were 0.7%−4.2%. Moreover, the *An. barbirostris* specimen (sample 10.2 – clade VIII) from Magelang-Central Java was also identified as a distinct species of the *An. barbirostris* complex in Indonesia. In comparison with other members in this study, the Magelang-Central Java ITS2 sequences displayed distances of 3.5%, 1.0%, 3.1–4.3%, and 4.0–4.5% with *An. barbirostris* from South Kalimantan (EU812759- EU812760 – clade I), Flores-East Nusa Tenggara (sample 9.2 – clade IX), Sulawesi (samples 1.2, 2.2, 4.2, 5.2, 6.2 – clade VI), and from Thailand (EU812761-EU812764 – clade I) respectively. The current study also suggests the presence of an additional new distinct population, i.e., *An. barbirostris* from Flores-East Nusa Tenggara (sample 9.2 – clade IX) identified as being relatively closely related to *An. barbirostris* k2 and k3 from South Kalimantan (EU812759-EU812760), although genetically distinct by 2.4% from them (Fig 2).

Species delimitation analysis using both a Maximum Likelihood and a Bayesian approach indicated the presence of four main populations corresponding to the four species previously described in Indonesia [3,8,16–18] (S1 Table, S1 Fig). They were identified with supports of 0.859, 0.926, 0.940 and 0.948. However, the species delimitation analysis also concluded on the presence of up to 9 more populations. This organization matches to that described using the phylogenetic analysis, i.e., four main populations A, B, C, and D, some of which include distinct subpopulations (S1 Table, S1 Fig). Population A (support 0.859) corresponds to *An. wejchoochotei* or clade V, which includes *An. barbirostris* from Manado and *An. campestris*-like from Thailand. Population B (support 0.926) corresponds to *An. saeungae* or clade IV from Thailand and Indonesia. Population C (support 0.940) comprises the Indonesian clades II, which is *An. vanderwulpi* and clade VII from Sumatra, as well as *An. barbirostris* sequences from Thailand. Population D was the most diverse and widely distributed one, comprising clades I or *An. barbirostris* s.s., VI, VIII, and IX. Clade III, which corresponds to *An. dissidens* has not been found in Indonesia, but only in Thailand.

### Phylogenetic analysis of the Cytochrome Oxidase I gene (COI)

**Fig 3:**
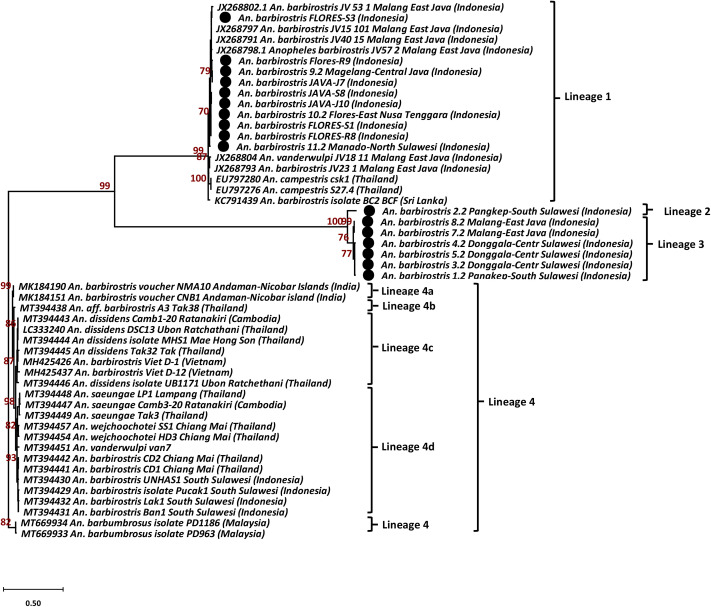
Phylogenetic analysis of the COI sequences. Tree rooted using *Anopheles barbumbrosus* as outgroup. All field collected samples are identified with black dot. Phylogenetic trees were constructed with the General Time Reversible (GTR)+1 model and constructed in Mega X. Bootstraps were tested with 1,000 replicates to assess the reliability of the trees.

The analysis of the COI sequences to identify the maternal lineage revealed that all samples and references from the GenBank belonged to three lineages of the *An. barbirostris* complex (Fig 3). Lineage 1 comprised two GenBank sequences of *An. campestris* from Thailand (EU797276, EU797280), one *An. barbirostris* from Sri Lanka (KC791439), 5 GenBank sequences of *An. barbirostris* from Malang-East Java (JX268791, JX268793, JX268797-JX268798, JX268802), one GenBank sequence of *An. vanderwulpi* from Malang-East Java (JX268804), and also included five different sequences of 106 specimens of An. barbirostris from Flores-East Nusa Tenggara (FLORES-S1, FLORES-S3, FLORES-R8, FLORES-R9, 9.2), four from Central Java (10.2, JAVA-J7, JAVA-S8, JAVA-J10), and one from Manado-North Sulawesi (11.2).

Lineage 2 consists of only one specimen of *An. barbirostris* from Pangkep-South Sulawesi (sample 2.2). While six specimens of *An. barbirostris* from Malang-East Java (samples 7.2, 8.2), Donggala-Central Sulawesi (samples 3.2, 4.2, 5.2), and Pangkep-South Sulawesi (sample 1.2) formed lineage 3 (ML bootstrap value 100%).

A branch point pattern indicating lineage 4 is separated into four sub-lineages. Sub-lineage 4a included two GenBank sequences of *An. barbirostris* from the Andaman-Nicobar Islands in India (MK184151, MK184190). Sub-lineage 4b comprised only *An. aff. barbirostris* from Thailand (MT394438). While sub-lineage 4c consists of five GenBank sequences of *An. dissidens*, one from Cambodia (MT394443) and four from Thailand (MT394444, MT394445, MT394446, LC333240), as well as two GenBank sequences of *An. barbirostris* from Vietnam (MH425426, MH425437). Sub-lineages 4d includes GenBank sequences of *An. barbirostris* from Chiang Mai-Thailand (MT394441-MT394442) and South Sulawesi-Indonesia (MT394429-MT394432), *An. wejchoochotei* from Chiang Mai-Thailand (MT394454, MT394457), *An. saeungae* from Cambodia (MT394447) and Thailand (MT394448-MT394449)*,* and *An. vanderwulpi* var.7 (MT394451) from Java, Indonesia. Two sequences of *An. barbumbrosus* from GenBank (MT669933-MT669934), originated from Malaysia, were used as the outgroup for the rooted tree (Fig 3).

Indonesian specimens of *An. barbirostris* within the lineage 1 cluster displayed some genetic variability of their COI sequences. *An. barbirostris* from Malang-East Java (JX268791, JX268793, JX268797-JX268798, JX268802) presented a percentage of divergence ranging between 0.2%−0.7%, while genetic distances ranging between 0.6–0.8% were observed with *An. vanderwulpi* from Malang-East Java (JX268804). The COI sequence divergence of the Indonesian samples with *An. campestris* from Thailand (EU797276, EU797280) and *An. barbirostris* from Sri Lanka (KC791439) was 0.5% and 0.6–0.7%, respectively.

## Discussion

*Anopheles barbirostris* s.l. has been previously reported in Indonesia as a complex comprising four distinct populations to which species names have now been given, i.e. *An. barbirostris* s.s, *An. saeungae, An. vanderwulpi*, and *An. barbirostris* from Sulawesi [3,17,18]. However, this simply corresponds to the definition of a metapopulation or interfertile populations, in other words four species sharing most of their genetic background, but also displaying significantly different genotypic and phenotypic traits. The use of the COI and ITS2 markers, beyond being very common in databases, is that they offer a way to investigate the diversity and phylogenetic relations of samples. COI being a mitochondrial gene, it will provide information on the maternal lineages and an insight on whether the samples have a single origin (monophyletic) or instead multiple origins (polyphyletic). ITS2 is a highly conserved nuclear marker and being a non-coding sequence, it is not submitted to selective pressure. It is thus a good marker to assess the presence of separate species. In this work, we report that a total of eight distinct populations can be identified in Indonesia within this *An. barbirostris* metapopulation or complex as commonly referred to in the *Anopheles* classification [2]. *Anopheles wejchoochotei* is a new species record within the Indonesian *An. barbirostris* complex. Previously, this latter population from Manado in North Sulawesi was identified as *An. campestris*, although it is not considered a malaria and lymphatic filariasis vector [9,20,31,32]. Thus, while *An. wejchoochotei* had only been reported in Thailand [7,8,13], this study shows that its geographic distribution extends to Indonesia. Furthermore, two specimens of *An. barbirostris* collected from Malang City in East Java (samples 7.2, 8.2) have also been suspected to represent a separate population. They display their closest relationship to *An. vanderwulpi* compared to other members of the *An. barbirostris* complex. This study also revealed that four specimens of *An. barbirostris* from Sulawesi (Pangkep-South Sulawesi – samples 1.2, 2.2, Donggala-Central Sulawesi – samples 4.2, 5.2), and Semarang-Central Java (sample 6.2) formed a distinct clade VI from other *An. barbirostris* specimens from Thailand and Indonesia. They represent thus another distinct population within the *An. barbirostris* complex. As previously reported, *An. barbirostris* from Sulawesi is a distinct species within the complex [9,16,18]. The metapopulation structure is well supported by both Maximum Likelihood and Bayesian analyses of species delimitation. Other differing traits linked to the metapopulation structure correspond to variations in bionomics such as behavioral and biological traits as well as vectorial competence. These traits are essential to investigate for implementing efficient vector control strategies. In Sulawesi, *An. barbirostris* plays an important role as a malaria and lymphatic filariasis vector [9,22]. Garjito et al. in 2004 reported that *An. barbirostris* in Donggala, Central Sulawesi, typically feeds outdoors during the night, and female mosquitoes predominantly bite humans from early dusk until midnight [33]. Even though Lien et al. in 1977 reported an anthropophilic form of *An. barbirostris* in Sulawesi, further investigations are needed to describe the various biting behaviors in areas where distinct populations are found [34]. However, these analyses have not been addressed in this work, which was devoted to deciphering the diversity of the overall Barbirostris complex (or metapopulation) in Indonesia as a primary but essential step. The characterization of the bionomics of each population will be the next step, along with a more in-depth exploration of the population structures of the different *An. barbirostris* species and metapopulation in Indonesia, based on larger samples. In this work, a distinct population of *An. barbirostris* (sample 9.2) has also been identified from Magelang in Central Java, located in the Menoreh Hills, a place where a novel species of *An. maculatus* was previously found [35,36]. The existence of distinct populations of *An. barbirostris* in Magelang is inseparable from changes in the continental shelf (2.58 Myrs to 11,700 years ago) that occurred during the Pleistocene Epoch, which resulted in increased volcanism in Central and Eastern Java. Menoreh Hills have been previously reported as a separated and preserved area from surrounding volcanic destruction, which served as a relic forest refuge [35,37]. Although this population was not confirmed as a malaria vector in the Java Island, Widiarti et al. reported that *An. barbirostris* specimens from Purworejo, Menoreh Hills, were positive for *P. vivax* infections both by CSP-ELISA and PCR methods [38].

*Anopheles barbirostris* has been documented as a major vector for malaria and lymphatic filariasis in East Nusa Tenggara [21]. Both *P. falciparum* and *P. vivax* were detected in Flores and Adonara Island in the eastern Lesser Sundas [19,21]. This taxon has also been incriminated as vector of lymphatic filariasis in eastern Lesser Sundas (*Brugia timori*) [39]. The existence of eight distinct Barbirostris populations, recorded in this study, provides valuable information on the diversity of malaria and lymphatic filariasis vector species in Indonesia, particularly in East Nusa Tenggara.

Currently, Indonesia is on the way to eliminating lymphatic filariasis and malaria, which are targeted to be achieved by 2030. However, several regions still face problems related to the high transmission of lymphatic filariasis and malaria, especially in Sulawesi and East Nusa Tenggara where *An. barbirostris* acts as the main vector [32,40,41]. Until 2019, the progress rate of lymphatic filariasis elimination in the provinces of Central Sulawesi, Southeast Sulawesi, West Sulawesi, Gorontalo, and South Sulawesi was significant and reached 44% (4/9), 50% (6/12), 50% (2/4), 67% (4/6), and 75% (3/4) of all districts/municipalities, respectively. However, in the province of East Nusa Tenggara, the lymphatic filariasis elimination program has only reached 17% (3/18) of the total districts/municipalities in the area. On the contrary, North Sulawesi is the only province in Sulawesi where all districts/cities are non-endemic for lymphatic filariasis [32].

Moreover, the Ministry of Health of Indonesia also noted that the progress of malaria elimination in the provinces of Gorontalo, Central Sulawesi, North Sulawesi, Southeast Sulawesi, South Sulawesi, and West Sulawesi had reached 66.7% (4/6), 61.5% (8/13), 60% (9/15), 52.9% (9/17), 16.7% (4/24) and 16.7% (1/6) of all districts/municipalities, respectively. However, a total of 34 districts/municipalities in Sulawesi are still malaria endemic areas, of which 33 districts/municipalities have low endemicity, while one district (located in Southeast Sulawesi) is still a medium endemic area [41]. In 2023, Papua was the province where malaria is most endemic accounting for at least 39.17% of all confirmed malaria cases in Indonesia with an annual total of 163,962 cases. Beside Papua, Central Papua is also one of the highest priority regions or provinces for malaria control in Indonesia. Data from the Ministry of Health of Indonesia revealed that as many as 150,225 cases (35.89%) out of 418,546 malaria cases countrywide in 2023 were reported in this province [42]. The third highest malaria endemic area in Indonesia is West Papua. A total of 43,862 malaria cases (10.47%) were reported in 2023 in this province. East Nusa Tenggara is the highest malaria-endemic area outside Papua Island, with a total of 6,968 cases (1.66%) identified in the same year [42,44]. *Anopheles barbirostris* s.l. has been documented in East Nusa Tenggara, but has never been recorded in Papua and West Papua [9].

Over the years, *An. barbirostris* s.l. became one of the main targets for vector control of lymphatic filariasis and malaria in the Lesser Sunda region (mostly East Nusa Tenggara) and Sulawesi [1,9,21,26,34,40,43]. However, most studies on bionomics and vectorial capacity of *An. barbirostris* s.l. in these areas were done, prior to the implementation of molecular analysis techniques, and based on unreliable morphological identification [5,8]. As a consequence, the diversity of populations and species within the *An. barbirostris* complex was not recognized and vector control efforts were then equalized in a variety of habitats and endemic areas. In contrast, the diversity of bionomics of species within this complex has a real impact on malaria epidemiology, transmission, and control, including vectorial capacity, insecticide resistance, feeding preferences, and resting preferences. This is why vector control efforts were not optimally targeted and the disease transmission is still going on [8,43]. This work offers the opportunity to base bionomics and vector competence studies on well-characterized *An. barbirostris* populations and species.

## Conclusions

Prior to this study, *An. barbirostris* s.l. in Indonesia was considered as having four distinct species, i.e., *An. barbirostris* s.s. in Java and Kalimantan, *An. vanderwulpi* in Java and Sumatra*, An. saeungae* in Sumatra, and an unknown form of *An. barbirostris* in Sulawesi. This study revealed that *An. barbirostris* s.l. in Indonesia is a more complex metapopulation consisting of at least eight populations. The correct characterization of the target populations is essential to better understand the geographic distribution and respective role in disease transmission of each member of the *An. barbirostris* complex. This is particularly true in areas where malaria and lymphatic filariasis transmission are still active, as well as in low transmission areas where the diseases are difficult to eliminate.

## Supporting information

S1 Table Estimates of Evolutionary Divergence between Sequences of ITS2 sequences. The number of base differences per site between sequences are shown. This analysis involved 41 nucleotide sequences. Codon positions included were 1st + 2nd + 3rd+Noncoding. All ambiguous positions were removed for each sequence pair (pairwise deletion option). There was a total of 393 positions in the final dataset. Evolutionary analyzes were conducted in MEGA11 [1]. The presence of n/c in the results denotes cases in which it was not possible to estimate evolutionary distances.
(XLSX)

S1 Fig Species delimitation using Bayesian and Maximum Likelihood methods based on ITS2 sequences. a. Distribution best supported by Bayesian estimates. b. Distribution supported by Maximum Likelihood.
(PPTX)

## References

[1] Reid JA. Anopheline Mosquitoes of Malaya and Borneo. Studies from the Institute for Medical Research Malaysia. 1968. p. 390–450.

[2] Harbach R. Anopheles classification. Last update Jan 6, 2024. 2024. https://mosquito-taxonomic-inventory.myspecies.info/sites/mosquito-taxonomic-inventory.info/files/Anopheles%20classification_71.pdf

[3] Paredes-Esquivel C, Donnelly MJ, Harbach RE, Townson H. A molecular phylogeny of mosquitoes in the Anopheles barbirostris Subgroup reveals cryptic species: implications for identification of disease vectors. Mol Phylogenet Evol. 2009;50(1):141–51. doi: 10.1016/j.ympev.2008.10.011 19000771

[4] Somboon P, Saeung A, Saingamsook J, Namgay R, Harbach RE. A new species of the Anopheles barbirostris complex (Diptera: Culicidae) from Bhutan. J Med Entomol. 2024;61(2):377–88. doi: 10.1093/jme/tjad161 38180303

[5] Satoto T. Cryptic species within Anopheles barbirostris van der Wulp, 1884, inferred from nuclear and mitocondrial gene sequence variation. Liverpool: University of Liverpool; 2001.

[6] Somboon P, Wilai P, Saeung A, Saingamsook J, Harbach RE. The identity of Anopheles (Anopheles) barbirostris species A3 of the Barbirostris Complex (Diptera: Culicidae). Zootaxa. 2023;5353(1):96–100. doi: 10.11646/zootaxa.5353.1.8 38221418

[7] Taai K, Harbach RE. Systematics of the Anopheles barbirostrisspecies complex (Diptera: Culicidae: Anophelinae) in Thailand. Zool J Linn Soc. 2015;174(2):244–64. doi: 10.1111/zoj.12236

[8] Brosseau L, Udom C, Sukkanon C, Chareonviriyaphap T, Bangs MJ, Saeung A, et al. A multiplex PCR assay for the identification of five species of the Anopheles barbirostris complex in Thailand. Parasit Vectors. 2019;12(1):223. doi: 10.1186/s13071-019-3494-8 31088534 PMC6515612

[9] Elyazar IRF, Sinka ME, Gething PW, Tarmidzi SN, Surya A, Kusriastuti R, et al. The distribution and bionomics of anopheles malaria vector mosquitoes in Indonesia. Adv Parasitol. 2013;83:173–266. doi: 10.1016/B978-0-12-407705-8.00003-3 23876873

[10] Sinka ME, Bangs MJ, Manguin S, Chareonviriyaphap T, Patil AP, Temperley WH, et al. The dominant Anopheles vectors of human malaria in the Asia-Pacific region: occurrence data, distribution maps and bionomic précis. Parasit Vectors. 2011;4:89. doi: 10.1186/1756-3305-4-89 21612587 PMC3127851

[11] Wang Y, Xu J, Ma Y. Molecular characterization of cryptic species of Anopheles barbirostris van der Wulp in China. Parasit Vectors. 2014;7:592. doi: 10.1186/s13071-014-0592-5 25511420 PMC4274727

[12] World Health Organization. Anopheline species complexes in South and South-East Asia. New Delhi, India: SEARO WHO; 2007. http://203.90.70.117/PDS_DOCS/B2406.pdf

[13] Udom C, Thanispong K, Manguin S, Chareonviriyaphap T, Fungfuang W. Trophic Behavior and Species Diversity of the Anopheles barbirostris Complex (Diptera: Culicidae) in Thailand. J Med Entomol. 2021;58(6):2376–84. doi: 10.1093/jme/tjab067 33942866

[14] O’Connor CT, Sopa T. A checklist of the mosquitoes of Indonesia. 1981. http://oai.dtic.mil/oai/oai?verb=getRecord&metadataPrefix=html&identifier=ADA117969

[15] Sukowati S, Andris H, Sondakh JS. Penelitian spesies sibling nyamuk Anopheles barbirostris van der wulp di Indonesia. J Ekologi Kes. 2004;4:172–80.

[16] Davidson JR, Wahid I, Sudirman R, Makuru V, Hasan H, Arfah AM, et al. Comparative field evaluation of kelambu traps, barrier screens and barrier screens with eaves for longitudinal surveillance of adult Anopheles mosquitoes in Sulawesi, Indonesia. Parasit Vectors. 2019;12(1):399. doi: 10.1186/s13071-019-3649-7 31409374 PMC6693138

[17] Townson H, Dyer N, Mcalister E, Satoto TBT, Bangs MJ, Harbach RE. Systematics of Anopheles barbirostris van der Wulp and a sibling species of the Barbirostris Complex (Diptera: Culicidae) in eastern Java, Indonesia. Systematic Entomology. 2013;38(1):180–91. doi: 10.1111/j.1365-3113.2012.00653.x

[18] Wilai P, Namgay R, Made Ali RS, Saingamsook J, Saeung A, Junkum A, et al. A Multiplex PCR Based on Mitochondrial COI Sequences for Identification of Members of the Anopheles barbirostris Complex (Diptera: Culicidae) in Thailand and Other Countries in the Region. Insects. 2020;11(7):409. doi: 10.3390/insects11070409 32630637 PMC7412068

[19] Bangs MJ, Rusmiarto S. Malaria vector incrimination in Indonesia using CSP-ELISA from 1986 to 2007. Jakarta, Indonesia. 2007.

[20] Manguin S, Bangs MJ, Pothikasikorn J, Chareonviriyaphap T. Review on global co-transmission of human Plasmodium species and Wuchereria bancrofti by Anopheles mosquitoes. Infect Genet Evol. 2010;10(2):159–77. doi: 10.1016/j.meegid.2009.11.014 19941975

[21] Marwoto H, Atmosoedjono S, Dewi R. Incrimination of malaria vector in Flores, East Nusa Tenggara. Bull Health Res. 1992;20:43–9.

[22] Machsoes M. Anopheles barbirostris als malaria overbrenger in deresidentie Celebes. Geneesk T Ned Ind. 1939;79:2500–15.

[23] Setiyaningsih R, Yanti AO, Lasmiati M, Prihatin MT, Widiarti W, et al. Anopheles diversity in forest ecosystem and risk of malaria transmission in several provinces in Indonesia. Media Litbangkes. 2019;29:243–54.

[24] Marwoto H, Richie TI, Atmosoedjono S, Tuti S, Tumewu M. Local malaria transmission in Manado Municipality. Bull Health Res. 1996;24:60–8.

[25] Harbach RE, Baimai V, Sukowati S. Some observations on sympatric populations of the malaria vectors Anopheles leucosphyrus and Anopheles balabacensis in a village-forest setting in South Kalimantan. Southeast Asian J Trop Med Public Health. 1987;18(2):241–7. 3313741

[26] Atmosoedjono S, Partono F, Dennis DT, Purnomo. Anopheles barbirostris (Diptera: Culicidae) as a vector of the timor filaria on Flores Island: preliminary observations. J Med Entomol. 1977;13(4–5):611–3. doi: 10.1093/jmedent/13.4-5.611 15122

[27] Atomosoedjono S, Van Peenen PF, Putrali J. Anopheles barbirostris (Van der Wulp) still an efficient vector of Brugia malayi in Central Sulawesi (Celebes), Indonesia. Trans R Soc Trop Med Hyg. 1976;70(3):259. doi: 10.1016/0035-9203(76)90056-0 982523

[28] O’Connor C, Soepanto A. Illustrated key to female anophelines of Indonesia. Jakarta, Indonesia: Directorate of Communicable Disease, Ministry of Health; 1979.

[29] Rattanarithikul R, Harrison B, Harbach R, Panthusiri P, Coleman R. Illustrated keys to the mosquitoes of Thailand. IV. Anopheles. Southeast Asian J Trop Med Pub Health. 2006;37:1–128. 17262930

[30] Rueanghiran C, Apiwathnasorn C, Sangthong P, Samung Y, Ruangsittichai J. Utility of a set of conserved mitochondrial cytochrome oxidase subunit I gene primers for Mansonia annulata identification. Southeast Asian J Trop Med Public Health. 2011;42(6):1381–7. 22299406

[31] Manguin S, Garros C, Dusfour I, Harbach RE, Coosemans M. Bionomics, taxonomy, and distribution of the major malaria vector taxa of Anopheles subgenus Cellia in Southeast Asia: an updated review. Infect Genet Evol. 2008;8(4):489–503. doi: 10.1016/j.meegid.2007.11.004 18178531

[32] MoH Indonesia. Lymphatic filariasis situation in Indonesia. Jakarta, Indonesia: Center data and information, Ministry of Health of Indonesia; 2019.

[33] Garjito TJ, Wijaya YL, Khadijah S, Erlan A, et al. Bio-ecological study of Anopheles in east coast areas of Parigi-Moutong district, Central Sulawesi. Bull Health Res. 2004;32:49–61.

[34] Lien JC, Kawengian BA, Partono F, Lami B, Cross JH. A brief survey of the mosquitoes of South Sulawesi, Indonesia, with special reference to the identity of Anopheles barbirostris (Diptera: Culicidae) from the Margolembo area. J Med Entomol. 1977;13(6):719–27. doi: 10.1093/jmedent/13.6.719 18607

[35] Garjito TA, Widiastuti U, Mujiyono M, Prihatin MT, Widiarti W, Setyaningsih R, et al. Genetic homogeneity of Anopheles maculatus in Indonesia and origin of a novel species present in Central Java. Parasit Vectors. 2019;12(1):351. doi: 10.1186/s13071-019-3598-1 31307517 PMC6631912

[36] Ali RSM, Wahid I, Saeung A, Wannasan A, Harbach RE, Somboon P. Genetic and morphological evidence for a new species of the Maculatus Group of Anopheles subgenus Cellia (Diptera: Culicidae) in Java, Indonesia. Parasit Vectors. 2019;12(1):107. doi: 10.1186/s13071-019-3358-2 30871633 PMC6419379

[37] Bemmelen R. General geology of Indonesia and adjacent archipelagoes. The geology of Indonesia. Hague: Government Printing Office; 1949. p. 1–709.

[38] Widiarti SR, Mujiyono R, Priyanto H, Barudin K. Entomological study of malaria vectors in Purworejo, Central Java. Salatiga: IVRCRD Salatiga; 2019.

[39] Lim B, Kurniawan L, Sudomo M, Joesoef A. Status of Brugian filariasis research in Indonesia and future studies. Bull Health Res. 1985;13:31–55.

[40] MoH Indonesia. Malaria elimination policy and strategies in Indonesia. Jakarta, Indonesia: Sub Directorate Malaria MoH Indonesia; 2019.

[41] MoH Indonesia. Indonesia Malaria Report 2019. Jakarta, Indonesia: MoH Indonesia; 2020. https://drive.google.com/file/d/1I8gA2IEq3a1HFJByVPZ7BjhtH0SpTI9R/view

[42] MoH I. Malaria cases in Indonesia 2023. https://malaria.kemkes.go.id/case

[43] Hoedojo. Vectors of malaria and filariasis in Indonesia. Bull Health Res. 1989;17:181–90.

[44] Cooper RD, Waterson DGE, Frances SP, Beebe NW, Sweeney AW. Speciation and distribution of the members of the Anopheles punctulatus (Diptera: Culicidae) group in Papua New Guinea. J Med Entomol. 2002;39(1):16–27. doi: 10.1603/0022-2585-39.1.16 11931251

