# Supplementary figures and images for: Anopheles barbirostris in Indonesia: A more complex metapopulation than expected
Source: PLoS One. 2025 Nov 10;20(11):e0321707. doi: 10.1371/journal.pone.0321707 (PMC12599951; doi:10.1371/journal.pone.0321707)

## Slide 1
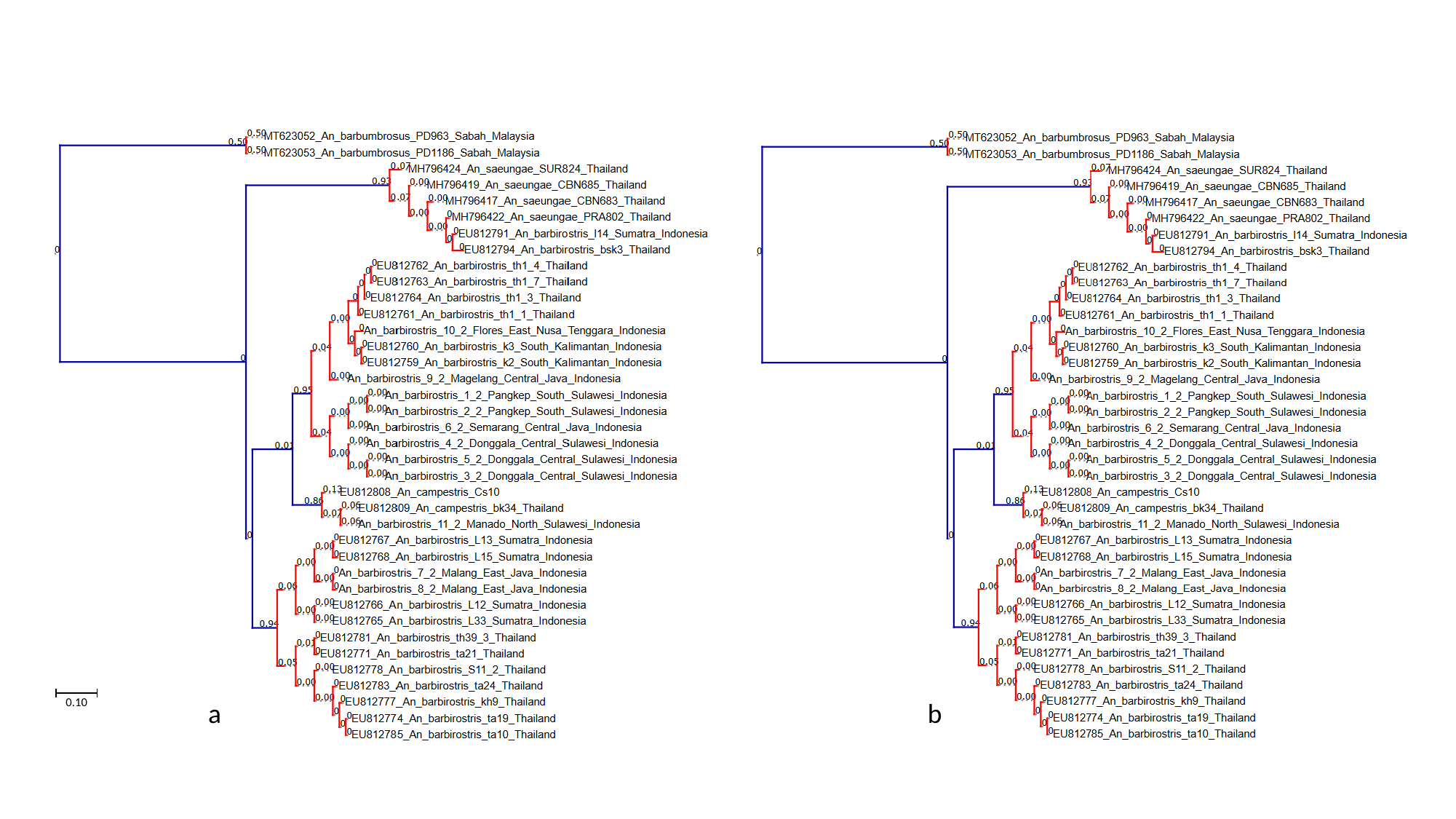

0.10
a
b

Supplement: S1 Fig — Distribution best supported by Bayesian estimates. b. Distribution supported by Maximum Likelihood. (PPTX) [file pone.0321707.s002.pptx]
